# A novel five-plex digital PCR assay for the simultaneous detection of murine pathogens: Sendai virus, reovirus, mouse parvoviruses, pneumonia virus of mice, and mouse hepatitis virus

**DOI:** 10.1128/spectrum.02548-25

**Published:** 2026-03-20

**Authors:** Tian Lan, Xin-yu Zhang, Yu-ying Li, Wei Chen, Lu-lu Xie, Yi-min Zhou, Yan Qin, Lin Zhou, Wen-chao Sun

**Affiliations:** 1 Wenzhou Key Laboratory for Virology and Immunology, Institute of Virology, Wenzhou University 26495, Wenzhou, China; 2 Agricultural College, Yanbian University 12396, Yanji, China; 3 College of Animal Sciences, Institute of Preventive Veterinary Medicine, Zhejiang University 12377, Hangzhou, China; Kwame Nkrumah University of Science and Technology, Kumasi, Ghana

**Keywords:** experimental animal viruses, Multiplex digital PCR, SeV, REO3, MPV, PVM, MHV

## Abstract

Animal viruses are significant factors that threaten the safety of laboratory animals and are related to the stability of laboratory animal quality. Early detection and diagnosis are crucial for effectively preventing and controlling viral contamination. Digital PCR technology is a novel detection technique that can greatly increase the sensitivity of detection methods. However, few reports exist on the use of digital PCR for detecting viruses in laboratory animals. Thus, in this study, we focused on five experimental animal viruses—Sendai virus (SeV), reovirus 3 (REO3), mouse parvovirus (MPV), pneumonia virus of mice (PVM), and mouse hepatitis virus (MHV)—that infect laboratory mice and rats relatively easily. A multiplex digital PCR assay using five distinct fluorescence signals (FAM, HEX, ROX, Cy5, and Cy3) was established for the simultaneous detection of viruses in experimental animals. Through a series of experiments, including optimization of the reaction system, specificity testing, sensitivity testing, and repeatability testing, a five-plex digital PCR method was developed. The results showed that five-plex dPCR could specifically detect target virus genes, with no specific amplification of VSV, SVA, MCMV, PPV, or PTV. The multiplex dPCR standard curves exhibited good linear relationships, with *R*^2^ values all above 0.99. Sensitivity experiments were conducted to determine the detection limits of the multiplex dPCR method for various viruses: REO3 at 1.21 × 10^1^ copies/μL, MPV at 1.27 × 10^1^ copies/μL, MHV at 0.87 × 10° copies/μL, SeV at 1.62 × 10° copies/μL, and PVM at 1.52 × 10^1^ copies/μL. Reproducibility experiments indicated that the within-batch and between-batch coefficients of variation for dPCR were 0.55–2.78% and 0–2.86%, respectively. The proposed method was proven to be reproducible. A total of 161 clinical samples were tested to evaluate the effectiveness of the method, with dPCR positivity rates for MHV, SeV, MPV, PVM, and REO3 of 0%, 16.8%, 3.1%, 3.1%, and 0.6%, respectively. Therefore, we successfully constructed a five-plex digital PCR system, laying a solid foundation for the rapid diagnostic detection of pathogens to improve health surveillance programs in laboratory animal facilities.

**IMPORTANCE:**

Digital PCR technology has been successfully applied to the detection of various viruses. Sendai virus (SeV), reovirus 3 (REO3), mouse parvovirus (MPV), pneumonia virus of mice (PVM), and mouse hepatitis virus (MHV) have seriously affected the health of experimental animals. To detect the five pathogens simultaneously, a multiplex digital PCR (dPCR) was established in this study. Furthermore, the five-plex digital PCR was rigorously evaluated, focusing on the limit of detection (LOD), sensitivity, specificity, and reproducibility. The results demonstrated excellent repeatability and specificity of the assay, which was successfully validated using 161 clinical samples. Our study provided a rapid and sensitive method for the simultaneous detection of five major viral pathogens affecting experimental animals.

## INTRODUCTION

Laboratory animals play important roles in the medical treatment of disease models and infectious disease models (1). However, experimental animal viruses, which are significant factors that threaten the quality of laboratory animals, have also garnered widespread attention (2). The presence of viruses not only endangers the health of laboratory animals but can also be transmitted to researchers during experiments, posing a potential health risk. Sendai virus (SeV) is a negative-sense RNA enveloped virus belonging to the *Paramyxoviridae* family and the *Respirovirus* genus (3). SeV is among the few viruses capable of causing clinical symptoms in immunocompetent animals, manifesting as pneumonia in infected mice, with clinical signs including respiratory distress, teeth chattering, and mortality in young animals (4). Reovirus 3 (REO-3) is a double-stranded RNA virus of the *Reoviridae* family, and REO3 infection in young mice results in symptoms such as stunted growth, diarrhea, oily coats, jaundice, and acute diffuse encephalitis (5). REO3 infection can affect cytokine levels, bacterial clearance from the lungs, tumor engraftment, and liver function in experimental animals (6, 7). Reovirus is also associated with various human diseases, including gastroenteritis, malabsorption, and hepatitis. Some studies have revealed a link between REO3 and neonatal biliary atresia (8). Pneumonia virus of mice (PVM) is a virus of the *Paramyxoviridae* family, subfamily *Pneumovirinae*, and is an enveloped, negative-sense, single-stranded RNA virus (9). PVM is among the numerous viral pathogens monitored in commercial and research rodent populations (10). Intense replication of PVM occurs in bronchial epithelial cells, which can lead to pulmonary edema, impaired respiratory function, and death (11). Mouse hepatitis virus (MHV) belongs to the *Coronaviridae* family and is an enveloped, positive-sense, single-stranded RNA virus (12). MHV causes a variety of diseases in susceptible rodents, including enteritis, hepatitis, and demyelinating encephalomyelitis (13). MHV-3 infection leads to severe hepatitis in mice, whereas infection with the neurotropic JHM strain of MHV causes acute encephalitis, which subsequently results in chronic demyelination to varying degrees among survivors (14). The virus is difficult to clear from the central nervous system (CNS), effectively resulting in persistent infection (15, 16). Mouse parvoviruses (MPVs) belong to the *Parvoviridae* family of nonenveloped DNA viruses (17). No clinical diseases or pathological lesions have been observed in terms of histology in mice experimentally or naturally infected with MPVs. Even in newborn and immunocompromised animals, the infection remains subclinical (18, 19). MPV-1 infection disrupts immune function, thereby affecting immunological studies in mice (17).

In recent years, the widespread use of animal models has led to a heightened focus on biosecurity practices and health monitoring of laboratory animals (20). Previous research has revealed the presence of various pathogenic contaminants in laboratories (21). A report from 1988 indicated that many commercially available rodents were seropositive for MHV and SeV. Sendai virus was prevalent in rat populations from 1988 to 1990, with up to 30% testing positive, but it decreased to 5.9% by 1996–1997; in contrast, the morbidity rate of rats infected with MPV remained stable or increased. In mice, the prevalence of contagious pathogens such as MHV is stable, whereas the prevalence of less transmissible pathogens such as PVM is significantly decreased (22). Data from 2003 revealed that MPV and MHV were the most common viruses, with MPV being the most prevalent in rats, followed by PVM and Theiler’s mouse encephalomyelitis virus (TMEV) (23). A three-decade surveillance in Western Europe published in 2006 indicated that MHV remained the most common virus in mice (24). In Taiwan in 2007, more than 20% of mice tested positive for MPV, MHV, and TMEV (25). A 2009 paper indicated that in European and North American laboratory mice, murine norovirus (MNV) was the most common virus, with a prevalence of 32%, which was much higher than that of other viruses. The prevalence of other viruses was lower, with MPV at 1.86%, MHV at 1.59%, and both PVM and REO-3 at 0.01%, while SeV was not detected (26). A 2011 study in Australia revealed that between 2004 and 2009, MNV was the most common virus (25.92%), followed by MHV, the second most prevalent virus in mice (3.86%) (27). Owing to strict biosafety practices, research results reported in 2023 revealed that many pathogens, including previously common pathogens, such as Sendai virus (0%), PVM (0.08%), and MHV (0.3%), decreased to less than 1% (28). These findings highlight the importance of proactive prevention and remediation. Even though many classic sources of infection are now very rare, testing for these infections should not be completely discontinued. These viruses may still exist in laboratory refrigerators and could lead to contamination. Preventing viral infections in experimental mice is vital for research, which underscores the importance of health monitoring (HM) (29). Consequently, a rapid, accurate, and cost-effective detection method is urgently needed.

Accurate viral detection is critical for ensuring experimental validity, particularly in animal models susceptible to covert infections. Before the emergence of nucleic acid testing (NAT), techniques such as cell culture, electron microscopy, complement fixation, agglutination tests, and immunological assays were widely used (30). However, these methods are time-consuming and have low sensitivity and accuracy, and they have been largely replaced by NAT tests (such as PCR) (31). Five experimental animal viruses (SeV, REO3, MPV, PVM, and MHV) are usually diagnosed through serological methods (ELISA and IFA) and can also be detected using PCR (32, 33). However, currently, no efficient method is available that can simultaneously detect multiple viruses. Digital PCR (dPCR) is a novel gene detection tool that has emerged in recent years. In dPCR, the sample to be tested is divided into multiple independent reaction units for PCR amplification (34). By analyzing the fluorescence signals and the Poisson distributions of positive and negative droplets, absolute quantification of target molecules can be achieved without relying on standard curves. dPCR offers high robustness, accuracy, and sensitivity (35). We aimed to establish a multiplex dPCR platform targeting SeV, REO3, MPV, PVM, and MHV to address the unmet need for the simultaneous detection of these pathogens. In this study, we developed a new method using multiplex dPCR technology with five different fluorescence signals (FAM, HEX, ROX, Cy5, and Cy3) for the simultaneous detection of these five viruses to which experimental animals are susceptible. Specificity tests, sensitivity tests, and reproducibility tests were conducted. The results indicate that both multiplex dPCRs exhibit good specificity, sensitivity, and reproducibility, with standard curves showing a strong linear relationship. We successfully established multiplex digital PCR for the detection of SeV, REO3, MPV, PVM, and MHV, providing technical methods for the clinical prevention and control of these pathogens.

## MATERIALS AND METHODS

### Main reagents, equipment, and virus preservation

The experiments were performed using a QIAcuity One 5-plex instrument (QIAGEN, Germany) and a real-time fluorescence quantitative PCR analyzer (BIOER, China). The QIAcuity Nanoplate 26k 24-well plate and QIAcuity Probe PCR Kit (250101) were purchased from QIAGEN, and Tag Pro HS Universal U+ Probe Master Mix (QN114-01) was acquired from Vazyme (China). The restriction enzymes BamHI (FD0054) and HindIII (FD0505) were both purchased from Thermo Fisher. PrimeScript RT Master Mix (RR036A) was purchased from Takara. The strains and genomes of vesicular stomatitis virus (VSV), Senecavirus A (SVA), porcine teschovirus (PTV), porcine circovirus (PCV2), porcine parvovirus (PPV), mouse cytomegalovirus (MCMV), PVM, MHV, REO3, and SeV are preserved in the Laboratory of Wenzhou University. Before use, the samples were stored at −80°C.

### Plasmid construction and primer design

The conserved genes selected as detection targets in this study included the nucleocapsid proteins of mouse hepatitis virus (FJ647223.1), Sendai virus (NC_001552.1), and pneumonia virus of mice (NC_006579.1); the outer capsid protein of Reovirus Type 3 (M20161.1); and the VP1 protein of mouse parvoviruses (DQ196319.1). The target genes were synthesized by Sangon Biotech (China) and ligated into the pUC57 vector to obtain approximately 4 μg of recombinant plasmid.

Following the selection of conserved regions in each target gene through sequence alignment, primers and probes were designed. These sequences were validated using the free online tool provided by Vazyme (evaluating key parameters, including complementary base pairing, hairpin structures, and Tm values). The fluorescence and quencher groups were selected according to the QIAcuity One 5-plex user manual. All primers and probes were synthesized by Tsingke (China) with purification by PAGE for primers and HPLC for probes. Detailed information on the primers and probes is listed in Table 1.

**TABLE 1:** Primers and TaqMan probes^*a*^

Name	Sequence (5′– 3′)	Product length (bp)
MPV-F	CTCTGGACTCTAACAACATA	104
MPV-R	GTACCTGTAAGGACTTGG	
MPV-P	HEX-CACACCAGCAACAGACAACCAA-BHQ1	
MHV-F	CTTGGAACTAGTGATCCA	145
MHV-R	ACTGCAATTCATACACATC	
MHV-P	CY5-TCATCAGCACCACCAGAGTTCTT-BHQ3	
REO3-F	CCAACGAATATGACACAG	125
REO3-R	CTACCTATCAAGTCTGCA	
REO3-P	FAM-ACTACTTCACTCACTACCGCACC-BHQ1	
SeV-F	GCAGACAAGTTATCCATTG	116
SeV-R	GGACTACTGTAAGCCATG	
SeV-P	CY3-ACTCATTAGACACAGATAAGCAGCACT-BHQ2	
PVM-F	AGGACAAGTTATGCTGAG	125
PVM-R	CTTGCTTCTGAGCATATTC	
PVM-P	ROX-AACCTCAACCACCTGTTCCATCT-BHQ2	

^*a*^ SEV, Sendai virus; REO3, reovirus type 3; MPV, mouse parvovirus; PVM, pneumonia virus of mice; MHV, mouse hepatitis virus.

### Optimization of dPCR

Plasmids diluted with ddH_2_O were mixed and stored in aliquots in a −80°C freezer. Copy numbers were calculated using the following formula: (plasmid concentration [ng] × 6.02 × 10^23^)/(genome length × 10^9^ × 660 Da/bp). After the five plasmids (SeV, REO3, MPV, PVM, and MHV) were mixed together, they were diluted in a 10-fold gradient to a concentration of 10^−1^ copies/μL. The reaction system and reaction conditions were initially explored using qPCR and needed to be optimized for dPCR. The system was prepared in PCR tubes according to the instructions of the QIAcuity Probe PCR Kit, and the restriction enzymes BamHI and HindIII were added to improve dPCR efficiency. The 40 µL dPCR system consisted of a 4 × QIAcuity Probe PCR Kit (10 µL), primers, probes, restriction enzymes (0.5 µL), the DNA/cDNA template, and ddH_2_O. Based on the requirements for QIAGEN proprietary PCR enzymes with conditions configured according to the manufacturer’s specifications, crystal dPCR was performed with the following initial steps: initial heat activation at 95°C for 2 min, followed by 45 cycles of denaturation at 95°C for 15 s and combined annealing/extension for 30 s. The reaction conditions, including the probe concentration, primer concentration, and annealing temperature, were optimized using the gradient method. The experiments were repeated for each sample and combination. The no-template control (NTC) was not used as a negative control.

### Standard curve and sensitivity assay of dPCR and qPCR

Five gradient concentrations (10^1^–10^5^ copies/μL) of five mixed plasmid standard samples were used as templates to construct the standard curve for the dPCR method. dPCR was performed using the optimized reaction system and conditions. Owing to the limitations of the fluorescence channel wavelength of the instruments from BIOER, the quintuple PCR was divided into triple PCR and double PCR for validation to establish the standard curve for the qPCR method. Standard plasmid samples mixed separately were diluted sequentially in a 10-fold gradient until 10^2^ copies/μL were obtained. Five gradient concentrations of standard plasmid samples ranging from 10^2^ to 10^6^ copies/μL were used as templates. In accordance with the instructions of the Tag Pro HS Universal U+ Probe Master Mix, qPCR was conducted using the optimized reaction system and conditions. The total reaction system of the multiplex qPCR was 20 μL, consisting of 10 μL of 2×Taq Pro HS Universal U+ Probe Master Mix, with concentrations of 0.4 μmol/L for the upstream and downstream primers of MPV, MHV, and REO3 (or SeV and PVM), and 0.5 μmol/L for each probe. The concentrations of the MPV, MHV, and REO3 (or SeV and PVM) mixed standard samples were the same as those used for dPCR, and ddH_2_O was added to bring the volume to 20 μL. The reaction program was set as follows: 95°C for 30 s, followed by 45 cycles of 95°C for 10 s and 58°C for 30 s. Each concentration was tested three times, and nuclease-free water was used as a negative control. A standard curve was plotted with the logarithmic value of the plasmid standard copy number on the X-axis and the average value of the corresponding detection concentration (qPCR) or the average number of threshold cycles (Ct) (dPCR) on the Y-axis to calculate the formula and correlation coefficient.

### Specificity and repeatability of dPCR and qPCR

The specificity of dPCR and qPCR was tested separately. SeV, MHV, REO3, SeV, PVM, MCMV, VSV, SVA, PCV2, and PTV were reverse-transcribed to generate cDNAs using the PrimeScript RT‒PCR Kit (TaKaRa, RR014A). The SeV, MHV, REO3, SeV, and PVM cDNAs and the positive MPV plasmids were mixed for dPCR and qPCR experiments as needed and tested according to the optimized system to validate specificity. RNase-free water was used as a negative control.

In the repeatability tests of the dPCR and qPCR systems, 10^3^–10^5^ (dPCR) and 10^4^–10^6^ (qPCR) copies/μL were used as templates for multiplex dPCR amplification to assess detection reproducibility. All the samples were subjected to three sequential 10-fold dilutions to ensure intrabatch repeatability.

### Evaluating the dPCR and qPCR assays with test samples

A total of 161 samples (anal and nasal swabs from 161 individual mice) collected from mice housed in nonbarrier and barrier environments were analyzed using established dPCR and qPCR assays. All the samples were resuspended in PBS and centrifuged at 12,000 rpm for 3 min to collect the supernatant for virus nucleic acid extraction.

Total cellular RNA was extracted using an RNA extraction kit (Sangon Biotech, B511311) according to the manufacturer’s instructions and then reverse-transcribed into cDNA using the PrimeScript RT‒PCR Kit (TaKaRa, RR014A). The established qPCR and crystal dPCR methods were used for detection. The positive detection rates were calculated to evaluate the sensitivity of the two methods for detection.

## RESULTS

### Optimization of the dPCR system

After measurement and calculation, the initial concentrations of the REO3, MPV, MHV, SeV, and PVM mixed plasmid standard samples were approximately 1.21 × 10^9^, 1.27 × 10^9^, 0.87 × 10^9^, 1.62 × 10^9^, and 1.52 × 10^9^ copies/μL, respectively. The mixed standard samples were diluted in a 10-fold gradient. The primer and probe reaction concentration screening results obtained using a mixed positive plasmid with a concentration of 10^5^ copies/μL as the template are shown in Table 2. When the total reaction volume was 40 μL, with a primer concentration of 0.375 μmol/L and a probe concentration of 0.250 μmol/L, the copy numbers for all five types were highest, and the 1D scatterplot performed excellently; hence, these concentrations were selected as the final primer and probe reaction concentrations. The annealing temperature for the dPCR was subsequently screened, and the results of the annealing temperature screen are shown in Table 3. When the annealing temperature was 58℃, the final copy numbers for all five primers were significantly greater than those at 59℃ and 60℃. The reaction program was established as follows: 95°C for 2 min; 95°C for 15 s; and 58°C for 30 s for 45 cycles. The exposure durations in the “Imaging” options for the green, yellow, orange, red, and crimson channels were set to 500, 400, 1,000, 200, and 325, respectively, with the gain options set at 5, 4, 6, 3, and 6. The five-plex digital PCR system was successfully optimized.

**TABLE 2:** Optimal concentrations of the primers and probes for the reaction^*a*^

Name	Concentration of the primers (μmol/L)	Mean ± SD (copies/μL)	
		Concentration of the probe (μmol/L)	
		0.250	0.500
REO3	0.125	12,161.33 ± 354.67	9,349.33 ± 89.33
	0.250	12,334.67 ± 230.67	8,018.67 ± 85.33
	0.375	13,228.00 ± 148.00	8,950.67 ± 146.67
	0.475	11,904.00 ± 44.00	8,745.33 ± 57.33
MPV	0.125	12,905.33 ± 170.67	11,876.00 ± 52.00
	0.250	12,956.00 ± 132.00	10,452.00 ± 32.00
	0.375	14,510.67 ± 122.67	12,741.33 ± 293.33
	0.475	13,024.00 ± 388.00	12,286.67 ± 65.33
MHV	0.125	4,577.33 ± 114.67	3,990.67 ± 38.67
	0.250	4,486.67±110.67	3,496.00 ± 104.00
	0.375	4,937.33 ± 73.33	4,228.00 ± 100.00
	0.475	4,400.00 ± 92.00	4,265.33 ± 101.33
SeV	0.125	23,033.33 ± 546.67	\
	0.250	23,276.00 ± 492.00	\
	0.375	25,410.67 ± 418.67	\
	0.475	22,826.67 ± 225.33	\
PVM	0.125	10,686.67 ± 70.67	10,064.00 ± 188.00
	0.250	11,057.33 ± 309.33	8,817.33 ± 182.67
	0.375	12,030.67 ± 114.67	10,680.00 ± 168.00
	0.475	10,924.00 ± 72.00	10,166.67 ± 154.67

^*a*^ \ indicates that the concentration exceeded the upper limit of quantification.

**TABLE 3:** Optimal annealing temperature

Melting temperature, Tm	Mean ± SD (copies/μL)		
	58°C	59°C	60°C
REO3	13,228.00 ± 148.00	12,674.67 ± 21.33	12,612.00 ± 288.00
MPV	14,510.67 ± 122.67	14,282.67 ± 165.33	14,068.00 ± 160.00
MHA	4,937.33 ± 73.33	4,816.00 ± 32.00	4,738.67 ± 46.67
SeV	25,410.67 ± 418.67	24,929.33 ± 350.67	24,365.33 ± 338.67
PVM	12,030.67 ± 26.67	11,633.33 ± 170.67	11,441.33 ± 114.67

### Construction of standard curves

**Fig 1:**
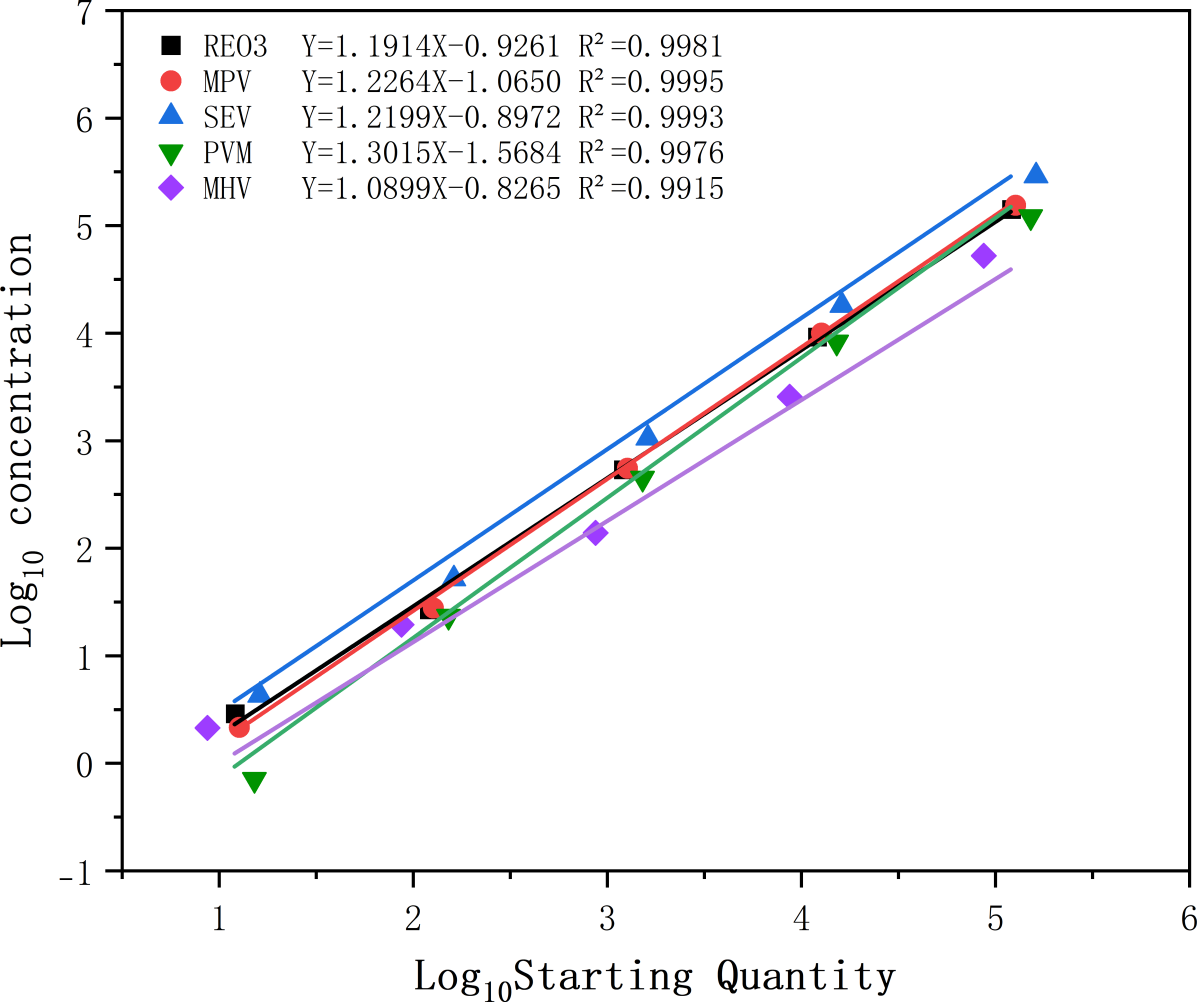
Standard curves for digital PCR. The standard curve was generated by plotting the concentration of the target DNA against the number of positive partitions. REO3: reovirus 3, MPV: mouse parvovirus, SEV: Sendai virus, PVM: pneumonia virus of mice, MHV: mouse hepatitis virus.

The concentration of the standard samples for multiplex dPCR, the copy number ranged from 10^−2^ to 10^5^ copies/μL, and the optimized program was used accordingly. The results of multiplex dPCR revealed that the *R*^2^ values of the standard curves for REO3, MPV, MHV, SeV, and PVM were 0.9981, 0.9995, 0.9993, 0.9976, and 0.9915, respectively (Fig. 1A). Good linearity was observed within the tested concentration range, but the performance of dPCR was still better than that of qPCR under the influence of potential factors (Fig. S1).

### Sensitivity analysis of multiplex dPCR

**Fig 2:**
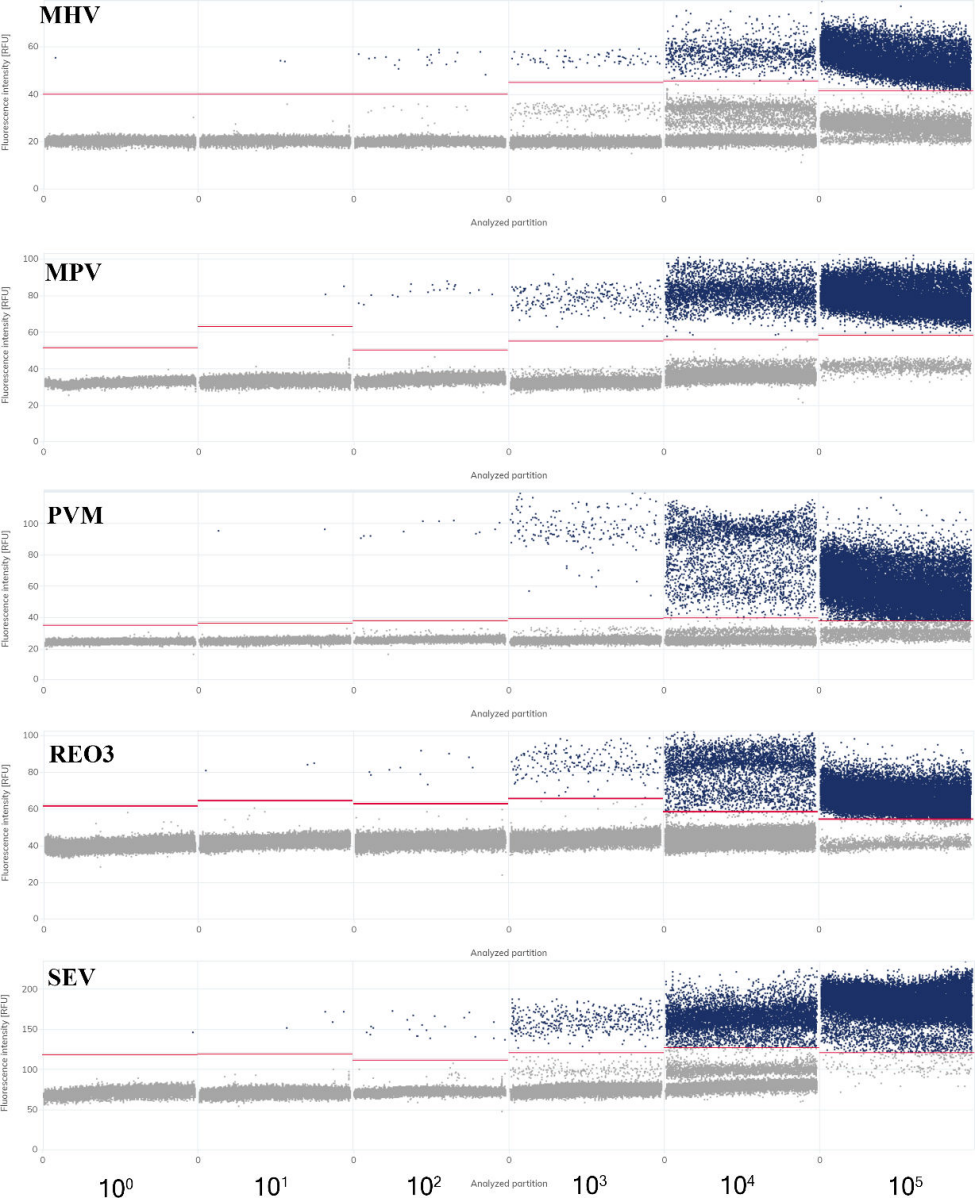
Detection limits of digital PCR. The lowest copy number of each standard sample detectable by multiplexed dPCR was between 10^0^ and 10^5^. NTC: no-template control.

The mixed standard plasmids were serially diluted 10-fold to obtain the lowest standard of 10^−2^ copies/μL for dPCR. The optimized dPCR system was applied for detection, with the criterion for positivity of positive droplets above the threshold line. The detection limits for dPCR were as follows: REO3, 1.21 × 10^1^ copies/μL; MPV, 1.27 × 10^1^ copies/μL; MHV, 0.87 × 10° copies/μL; SeV, 1.62 × 10° copies/μL; and PVM, 1.52 × 10^1^ copies/μL (Fig. 2). The dPCR detection method exhibited good sensitivity. Furthermore, although the sensitivity of multiplex qPCR was not as good as that of dPCR, its detection limit reached 0.87 × 10^1^ copies/μL (Fig. S2). Both dPCR and multiplex qPCR exhibited good sensitivity.

### Repeatability analysis of multiplex dPCR

In the repeatability tests of the dPCR systems, three concentration gradients of 10^3^–10^5^ (dPCR) were simultaneously tested. The coefficients of variation (CVs) for the intrabatch and interbatch data were calculated. The results of the dPCR experiments revealed that the minimum and maximum values of the intrabatch CV were 0.55% and 2.78%, respectively; the minimum and maximum values of the interbatch CV were 0% and 2.86%, respectively (Table 4). Detailed reproducibility data for the triplex and duplex PCR setups are available in the Supplementary Information (Tables S1 and S2). The repeatability of the dPCR and qPCR systems was good.

**TABLE 4:** Repeatability test of multiplex digital PCR

Plasmid (copies/μL)		Intra-assay variability		Inter-assay variability	
		Mean ± SD (copies/μL)	CV (%)	Mean ± SD (copies/μL)	CV (%)
REO3	10^3^	537.33 ± 10.67	1.99%	513.33 ± 14.67	2.86%
	10^4^	9,216.00 ± 132.00	1.43%	8,748.00 ± 120.00	1.37%
	10^5^	141,242.67 ± 3,358.67	2.38%	128,033.33 ± 3,629.33	2.83%
MPV	10^3^	552.00 ± 8.00	1.45%	569.33 ± 14.67	2.58%
	10^4^	9,977.33 ± 206.67	2.07%	9,652.00 ± 108.00	1.12%
	10^5^	154,410.67 ± 1,577.33	1.02%	140,372.00 ± 456.00	0.32%
MHV	10^3^	138.67 ± 2.67	1.92%	160.00 ± 0.00	0.00%
	10^4^	2,558.67 ± 69.33	2.71%	2,400.00 ± 8.00	0.33%
	10^5^	52,366.67 ± 289.33	0.55%	46,905.33 ± 497.33	1.06%
SeV	10^3^	1,054.67 ± 29.33	2.78%	1,034.67 ± 14.67	1.42%
	10^4^	18,061.33 ± 170.67	0.94%	17,081.33 ± 174.67	1.02%
	10^5^	289,733.33 ± 3,638.67	1.26%	306,816.00 ± 8,736.00	2.85%
PVM	10^3^	448.00 ± 8.00	1.79%	429.33 ± 9.33	2.17%
	10^4^	8,241.33 ± 133.33	1.22%	7,774.67 ± 93.33	1.20%
	10^5^	120,721.33 ± 2,217.33	1.84%	101,446.67 ± 1,650.67	1.23%

### Specificity analysis of multiplex dPCR

**Fig 3:**
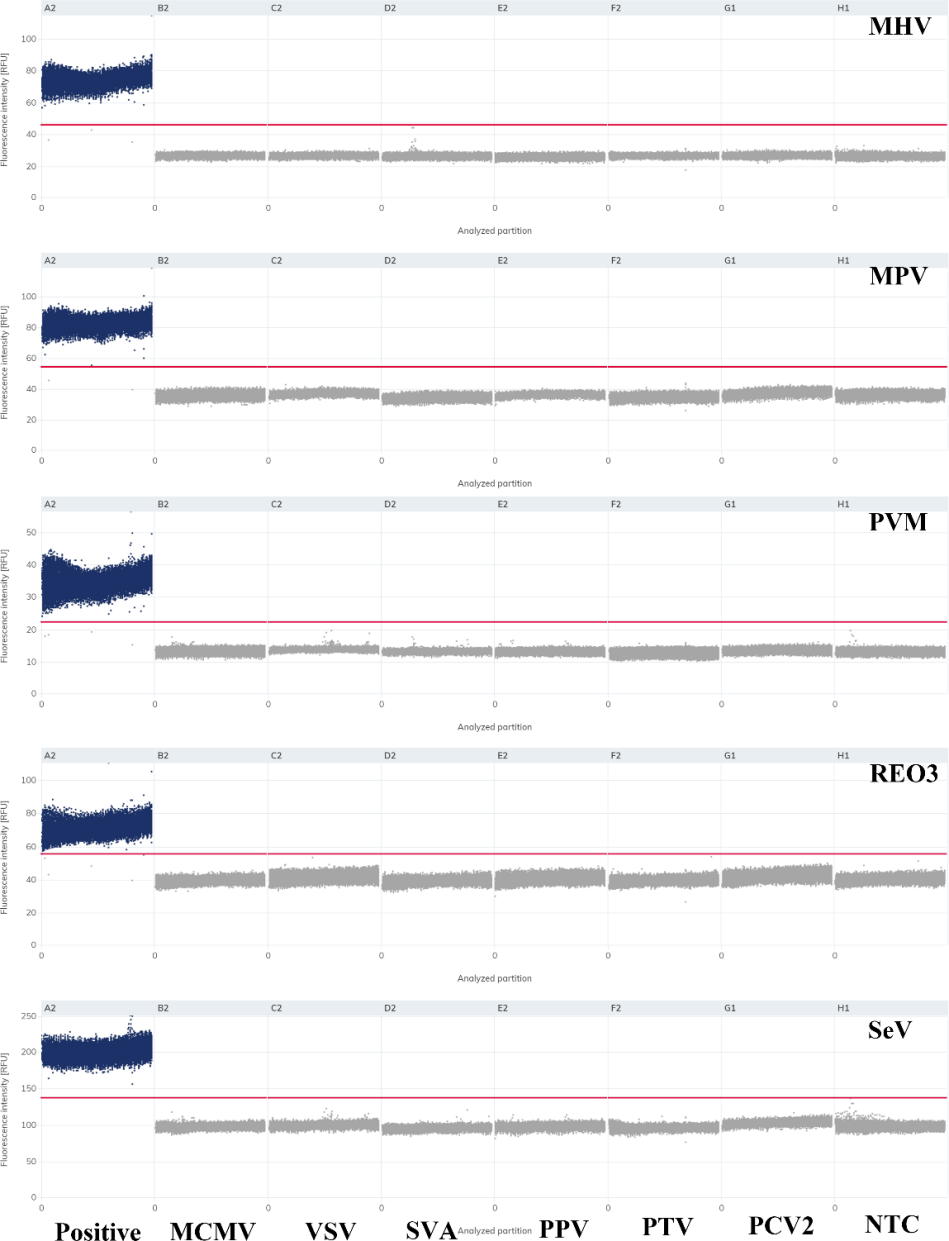
Specificity analysis of multiplex digital PCR. The specificity of multiplex dPCR is shown. Positive droplets (dPCRs) for MCMV, VSV, SVA, PPV, PCV2, and PTV are shown separately. All the samples are labeled in the figure. NTC refers to the no-template control, which yielded negative results with no positive droplets detected.

Following the validation of the method criteria, including sensitivity and intra-/inter-assay repeatability, the specificity of the multiplex dPCR assays was assessed. In the specificity assay of the dPCR method, in addition to the positive cDNAs for MHV, REO3, SeV, and PVM and the plasmids of MPV, no positive droplets were generated for MCMV, VSV, SVA, PPV, PCV2, PTV, and NTC, or only one positive droplet was generated (Fig. 3A). No nonspecific amplification curves were observed in the multiplex qPCR analysis (Fig. S3). These findings indicate that the detection method established in the present study has good specificity.

### Clinical tests of multiplex dPCR

In total, 161 samples consisting of mouse anal and nasal swabs were tested using multiplex dPCR. The criterion for judgment was more than three positive droplets in dPCR. The results of multiplex dPCR revealed positive detection rates for MHV, SeV, MPV, PVM, and REO3 of 0%, 16.8%, 3.1%, 3.1%, and 0.6%, respectively. During the testing process, cases of simultaneous infection with multiple viruses were found in the same clinical samples. The mixed infection rates of SeV and MPV, SeV and PVM, and SeV, MPV, and REO3 were 3.1% (5/161), 3.1% (5/161), and 0.6% (1/161), respectively. We presented the coinfected viruses in a table format, showing the viruses infected at the corresponding coordinates (Table 5), and observed that PVM and MPV were always coinfected with SeV. The positive detection rates of MHV, SeV, MPV, PVM, and REO3 using multiplex qPCR were 0%, 12.4%, 1.8%, 3.1%, and 0%, respectively (Table S3). A comparison of the two detection results is shown in Table 6.

**TABLE 5:** Clinical tests of multiplex digital PCR^*a*^

Positive samples	MHV	SeV	MPV	PVM	REO3	Sole viral infection
MHV	–	0	0	0	0	0
SeV	0	–	5	5	1	16
MPV	0	5	–	0	1	0
PVM	0	5	0	–	0	0
REO3	0	1	1	0	–	0

^*a*^ The table presents the combinations of viruses identified in the positive samples. The primary section of the table tabulates the occurrences of diverse viral codetections, representing cases of coinfection. The column labeled “Sole viral infection” on the right signifies the count of samples solely infected by a single virus. – indicates that the data point is not applicable.

**TABLE 6:** Differences in clinical tests

Detection method	Detection results (positive samples/total samples)				
	MHV	SeV	MPV	PVM	REO3
Multiplex dPCR	0/161	27/161	5/161	5/161	1/161
Multiplex qPCR	0/161	20/161	3/161	5/161	0/161

## DISCUSSION

Digital PCR (dPCR) represents a new generation of traditional quantitative polymerase chain reaction (qPCR), offering numerous advantages. Compared with real-time quantitative fluorescence PCR, digital PCR eliminates the need for a standard curve, thereby avoiding errors associated with sample concentration and offering greater accuracy (36, 37). dPCR has two notable features: compartmentalization and the collection of data from endpoint reactions. Each droplet has a particular encapsulated area that prevents cross‐contamination between micro bioreactors (38). Compartmentalization amplifies the signal of the target gene relative to that of other genes, thereby improving the signal-to-noise ratio; moreover, endpoint measurements are less dependent on reaction efficiency (39). Even when the same primers, probes, Taq polymerase, and reagents used in traditional PCR for the amplification of target DNA fragments are utilized, dPCR demonstrates significantly better sensitivity and reproducibility (40). Mismatched primers in qPCR notably reduce amplification efficiency, whereas dPCR shows increased resistance to mismatches (41, 42). Additionally, dPCR results in greater tolerance to PCR inhibitors (43, 44). In recent years, digital PCR has led to the development of multiple systems for detecting viruses. These systems can detect SARS-CoV-2 (45) and canine distemper virus (CDV) (46) individually. In a cohort of subjects with COVID-19 pneumonia whose diagnostic SARS-CoV-2 RT‒qPCR results were negative, 11 of the 18 cases were identified as false negatives. After the application of dPCR, the overall sensitivity of viral molecular detection increased from approximately 72% to 89% (45). The detection limit for CDV using dPCR is relatively low, measuring 3 copies/μL, which is lower than the previously reported range of 10^1^ to 10^2^ copies for qPCR. Compared with traditional diagnostic methods, the application of dPCR has led to a 25% increase in the detection rate of CDV-positive cases (46). Furthermore, triple digital PCR can be used to detect duck Tembusu virus (DTMUV), duck circovirus (DuCV), and new duck reovirus (NDRV) (47); similarly, it can also be used to detect African swine fever virus (ASFV), classical swine fever virus (CSFV), and porcine reproductive and respiratory syndrome virus (PRRSV) (48). Multiplex digital PCR, with its high sensitivity and multiplex detection capabilities (simultaneously identifying ASFV, CSFV, and PRRSV, or DTMUV, DuCV, and NDRV), can accurately detect low-concentration pathogens (such as early infections), significantly improving the efficiency of the diagnosis of mixed infections. It enables targeted clearance, reduces the risk of viral spread, and provides an effective tool for disease treatment and prevention.

In this study, multiplex digital PCR (dPCR) was developed to simultaneously detect five common pathogens: SeV, REO3, MPV, PVM, and MHV. Primers and probes targeting conserved sequences in viral genomes were designed, and different reaction conditions were optimized to evaluate the specificity, sensitivity, and reproducibility of this method. The results show that multiplex dPCR can be used to detect specific target viral genes without specifically amplifying the VSV, SVA, MCMV, PPV, PCV2, or PTV cDNA. Sensitivity tests revealed that the lowest detection limits of multiplex dPCR were as follows: REO3, 1.21 × 101 copies/μL; MPV, 1.27 × 101 copies/μL; MHV, 0.87 × 100 copies/μL; SeV, 1.62 × 100 copies/μL; and PVM, 1.52 × 101 copies/μL. Previous research has shown that fnRT‒PCR can detect as little as 10 fg of SeV RNA and 1 pg of PVM RNA (49). Both MHV RT-RPA and RT‒PCR present the same limit of detection, which is 4.45 × 10¹ copies/μL (50). The minimum concentration of MPV detectable by PCR-HRM is 10 copies/μL (51). Additionally, fnRT‒PCR for REO3 can detect less than 1 fg, while the limit of detection for RT‒LAMP is 4 fg/μL (52, 53). In comparison, the five-plex digital PCR system we constructed has a slightly better limit of detection than those used in previous studies. Reproducibility experiments demonstrate that the intrabatch and interbatch coefficients of variation for dPCR are 0.55%–2.78% and 0%–2.86%, respectively. These findings indicate that the method has good stability, ensuring the authenticity and reliability of the experimental research. The multiplex dPCR results revealed positive detection rates of 0%, 16.8%, 3.1%, 3.1%, and 0.6% for MHV, SeV, MPV, PVM, and REO3, respectively. The sample detection results indicate that compared with multiplex qPCR, multiplex dPCR has a slightly superior detection capability. However, owing to the drawbacks of dPCR, such as its limited reaction volume, low sample detection capacity, and high cost, its widespread clinical application is restricted. For instance, this method restricts its utility in situations that require swift and economical screening of numerous samples, such as mass population entry quarantine or routine screening of breeding collectives, for which qPCR proves to be a more cost-effective and efficient alternative. The dPCR is used for detecting extremely high precision requirements, with specific applications including monitoring precious samples, early infection diagnosis, virus load tracking, and confirming suspicious results. In particular, when the qPCR results are close to the threshold, dPCR can be used as the final determinant. Additionally, it can delve into complex infections and accurately quantify the load of different pathogens to elucidate their relationships. These drawbacks of dPCR will likely be addressed, and the method will be widely promoted in the field of microbiological research. The increasing interest in, and necessity for, transgenic animals, such as immunodeficient mice, along with their widespread sharing among academic institutions, has made rigorous pathogen testing before their release from isolation facilities an indispensable component of institutional health surveillance programs. The quintuple droplet digital polymerase chain reaction (dPCR) method developed in this research holds distinct significance in this context. Its exceptional sensitivity renders it well-suited for early infection detection in valuable animal strains, enabling precise interventions before the onset of disease outbreaks. This method serves as a robust protective measure for maintaining the health and security of animal colonies during quarantine, effectively preventing significant losses linked to pathogen dissemination.

## Data Availability

The data that support the findings of this study are available from the corresponding author upon reasonable request.

## SUPPLEMENTAL MATERIAL

Supplemental material spectrum.02548-25-s0001.docx Fig. S1 to S3; Tables S1 to S3.

ASM does not own the copyrights to Supplemental Material that may be linked to, or accessed through, an article. The authors have granted ASM a non-exclusive, world-wide license to publish the Supplemental Material files. Please contact the corresponding author directly for reuse.
