# Supplementary material for: A novel five-plex digital PCR assay for the simultaneous detection of murine pathogens: Sendai virus, reovirus, mouse parvoviruses, pneumonia virus of mice, and mouse hepatitis virus
Source: Microbiol Spectr. 2026 Mar 20;14(4):e02548-25. doi: 10.1128/spectrum.02548-25 (PMC13055384; doi:10.1128/spectrum.02548-25)
Supplement: Supplemental material — Fig. S1 to S3; Tables S1 to S3. [file spectrum.02548-25-s0001.docx]

| **Table S1.** **Repeatability test of Triple qPCR** | | | | | | | |
| --- | --- | --- | --- | --- | --- | --- | --- |
| Plasmid (copies/μL) | | Intra-assay variability | | | Interassay variability | | |
|  |  | mean (copies/μL) | SD | CV (%) | mean (copies/μL) | SD | CV (%) |
| REO3 | 10^6^ | 14.88 | 0.25 | 1.66% | 16.27 | 0.43 | 2.64% |
|  | 10^5^ | 19.02 | 0.50 | 2.63% | 19.65 | 0.32 | 1.61% |
|  | 10^4^ | 23.11 | 0.12 | 0.53% | 23.51 | 0.44 | 1.89% |
| MPV | 10^6^ | 15.89 | 0.16 | 0.99% | 15.94 | 0.16 | 1.02% |
|  | 10^5^ | 19.41 | 0.55 | 2.82% | 19.94 | 0.72 | 3.61% |
|  | 104 | 23.76 | 0.13 | 0.56% | 23.01 | 0.46 | 1.98% |
| MHV | 10^6^ | 17.95 | 0.36 | 1.99% | 18.39 | 0.15 | 0.83% |
|  | 105 | 21.30 | 0.09 | 0.41% | 21.45 | 0.08 | 0.39% |
|  | 10^4^ | 25.42 | 0.07 | 0.29% | 25.62 | 0.10 | 0.38% |

**qPCR repeatability analysis.** For the triple and double qPCR experiments, the minimum and maximum values of intragroup CVs were 0.29%-2.82% and 0.38%-3.61%, respectively. The minimum and maximum values of interbatch CV were 0.51%-3.19% and 1.17%-3.62%, respectively (Tables S1-S2).

**Table S2.** **Repeatability test of double qPCR**

| Plasmid (copies/μL) | | Intra-assay variability | | | Interassay variability | | |
| --- | --- | --- | --- | --- | --- | --- | --- |
|  |  | mean (copies/μL) | SD | CV (%) | mean (copies/μL) | SD | CV (%) |
| SeV | 10^5^ | 19.16 | 0.40 | 2.07% | 18.68 | 0.38 | 2.05% |
|  | 10^4^ | 23.00 | 0.12 | 0.51% | 22.55 | 0.26 | 1.17% |
|  | 10^3^ | 26.67 | 0.51 | 1.90% | 26.22 | 0.95 | 3.62% |
| PVM | 10^5^ | 19.14 | 0.12 | 0.64% | 18.92 | 0.43 | 2.26% |
|  | 10^4^ | 22.47 | 0.20 | 0.89% | 22.66 | 0.46 | 2.02% |
|  | 10^3^ | 27.46 | 0.88 | 3.19% | 28.84 | 0.39 | 1.35% |

**Clinical tests for qPCR** In total, 161 samples were tested using qPCR. The criteria for judgment were a qPCR cycle threshold of less than 35. The positive detection rates of MHV, SeV, MPV, PVM, and REO3 using multiplex qPCR were 0%, 12.4%, 1.8%, 3.1%, and 0%, respectively (Table S3). The combined infection rates of SeV and MPV and of SeV and PVM were 1.8% (5/161) and 3.1% (5/161), respectively. This table details the combinations of viruses detected in the positive samples. The main body of the table lists the number of samples with different viral codetections, reflecting instances of coinfection. The column on the right, labeled “single viral infection,” indicates the number of samples infected with only one virus.

| Positive samples | MHV | SeV | MPV | PVM | REO3 | Sole viral infection |
| --- | --- | --- | --- | --- | --- | --- |
| MHV | - | 0 | 0 | 0 | 0 | 0 |
| SeV | 0 | - | 3 | 5 | 0 | 12 |
| MPV | 0 | 3 | - | 0 | 0 | 0 |
| PVM | 0 | 5 | 0 | - | 0 | 0 |
| REO3 | 0 | 0 | 0 | 0 | - | 0 |

**Table S3.** **Clinical test for multiplex qPCR**

**Figure legends**

**
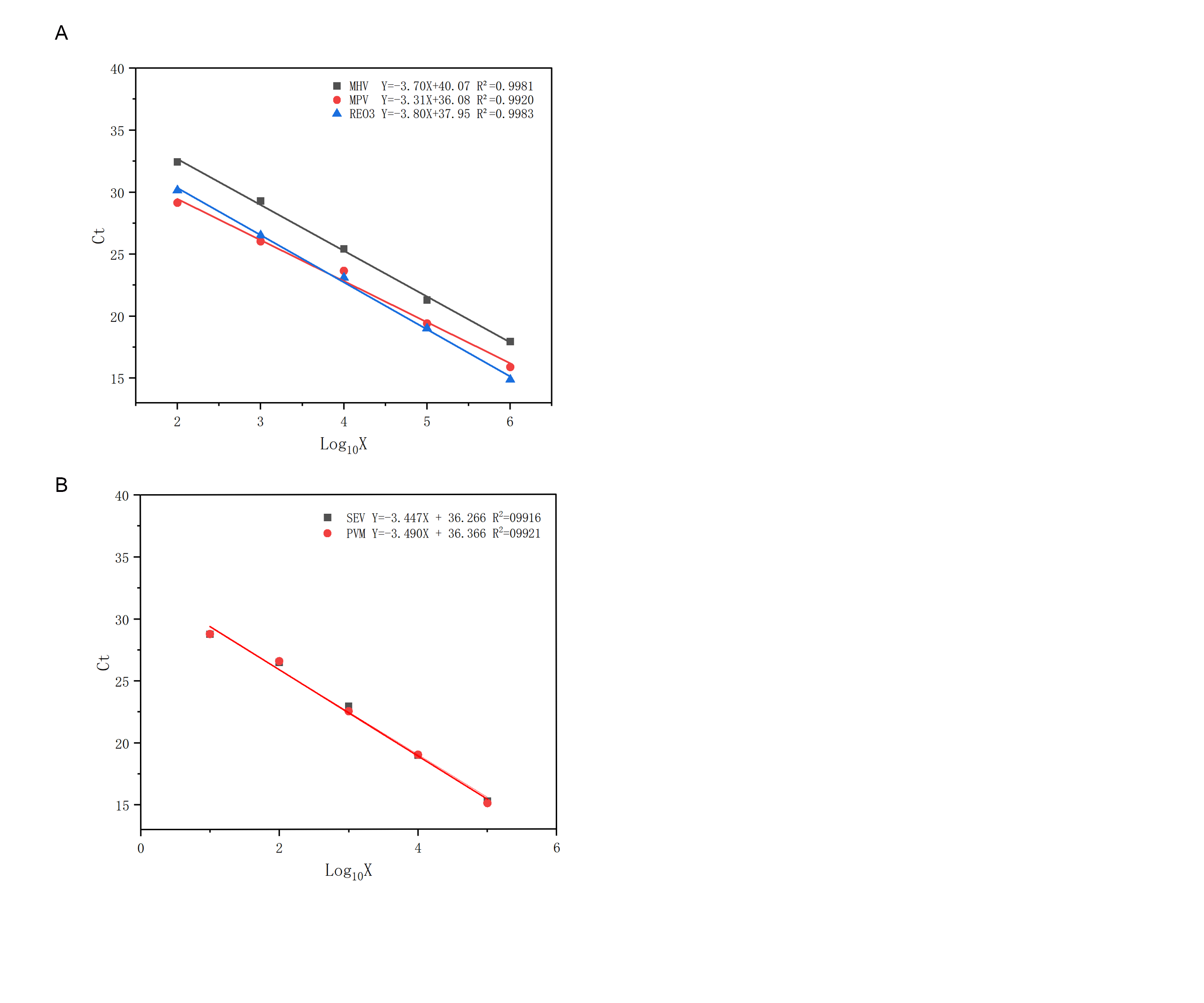
**

**Figure S1. Standard curves of qPCR.**

Figures S1A and S1B show the standard curves for triple qPCR and double qPCR, respectively. The concentrations of the standard plasmids for triple and double qPCR ranged from 10^2^ to 10^6^ copies/μL. The *R*^2^ values of the standard curves for triple qPCR of MPV, MHV, REO3 were 0.9920, 0.9981, 0.9983, respectively (Figure S1A), while for double qPCR of SeV and PVM, the R2 values were 0.9916 and 0.9921(Figure S1B).


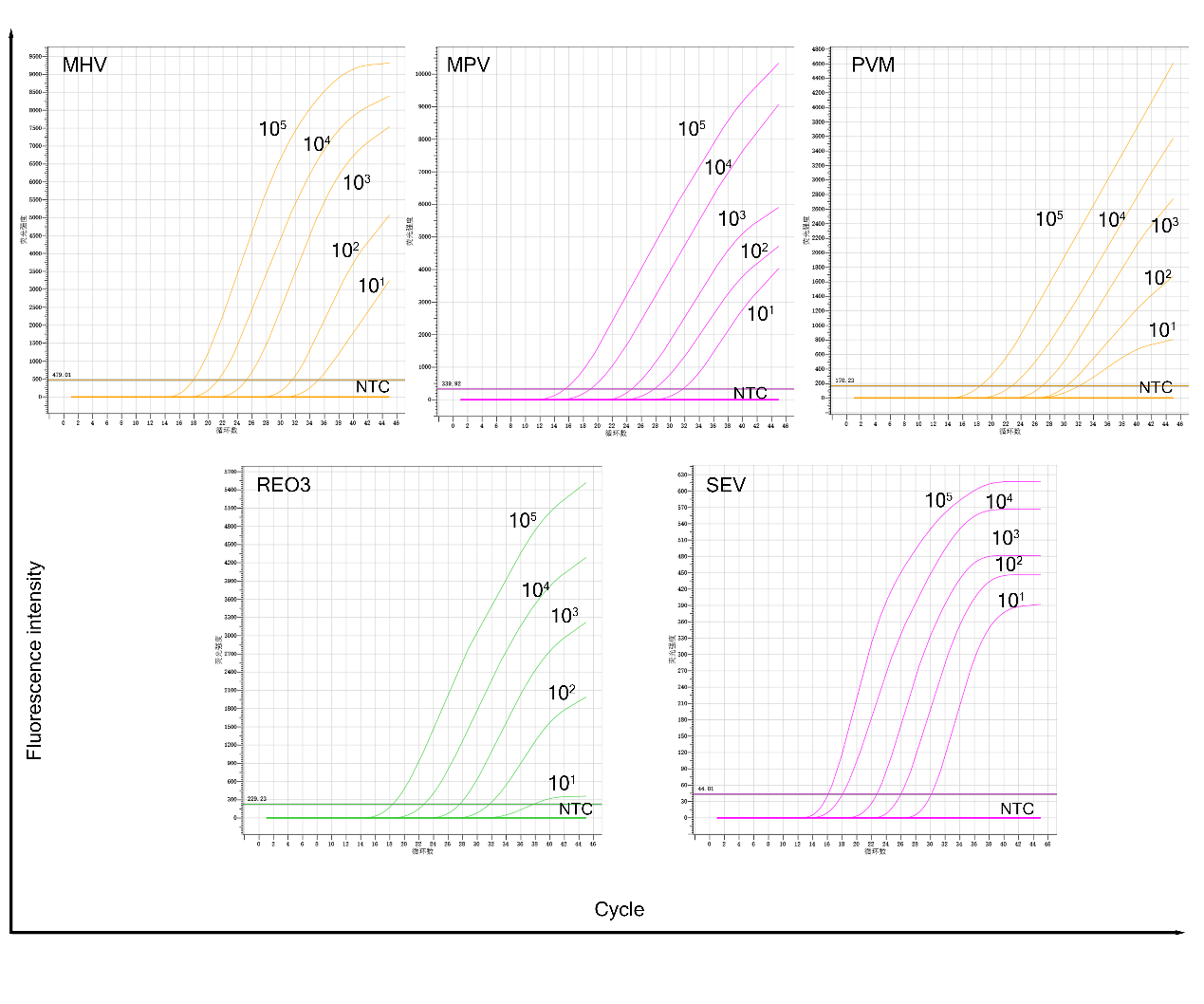
**Figure S2. Detection limits of qPCR.**

Figure S2 shows the lowest copy number of each standard sample detectable by qPCR between 10^1^ and 10^5^. In this case, 10^0^, 10^1^, 10^2^, 10^3^, 10^4^, and 10^5^ represent the copy numbers of the standard samples. NTC：No Template Control. The detection limits for qPCR were REO3 1.21×10^1^ copies/μL, MPV 1.27×10^1^ copies/μL, MHV 0.87×10^1^ copies/μL, SeV 1.62×10^1^ copies/μL, and PVM 1.52×10^1^ copies/μL


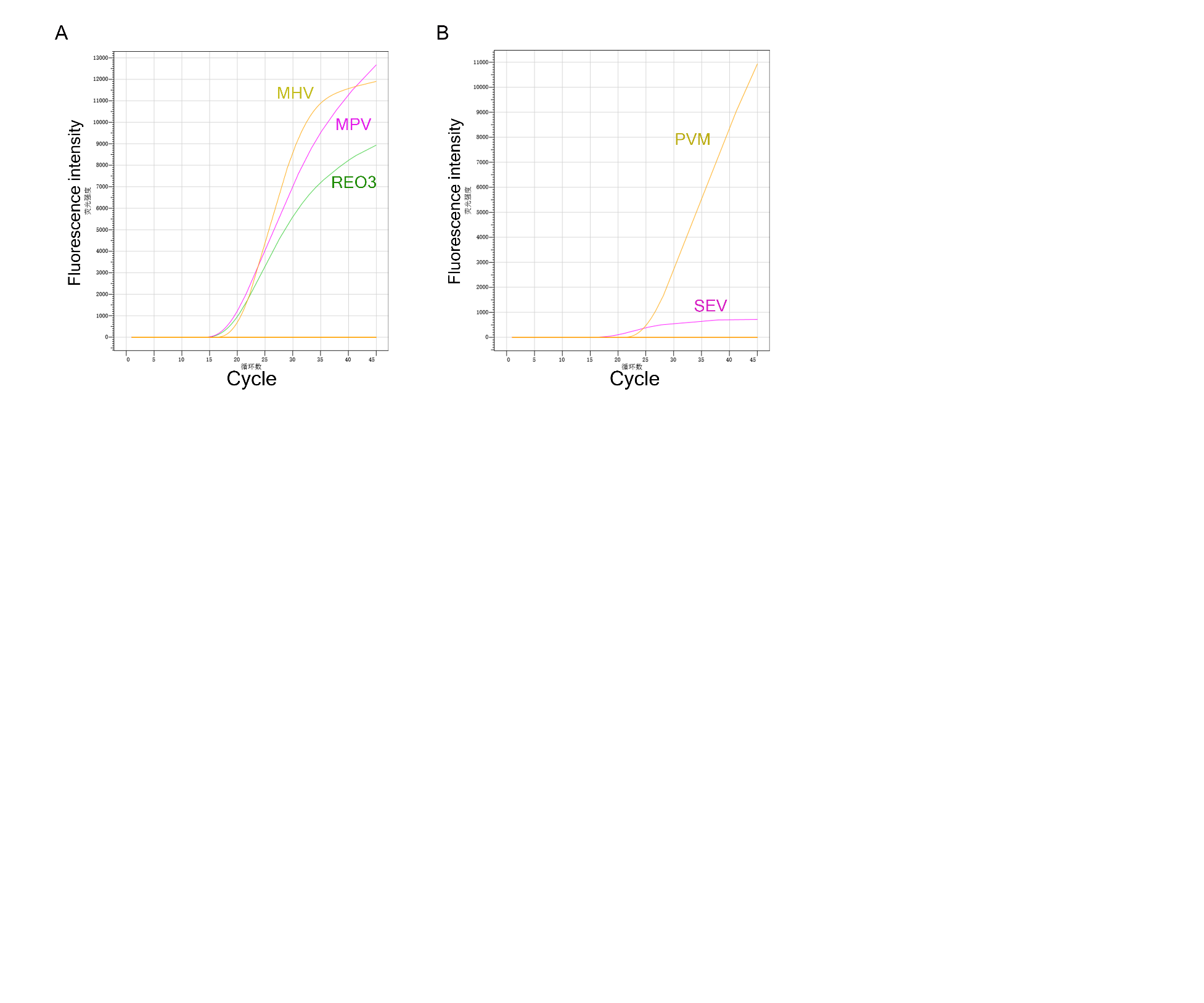


**Figure S3. Specificity analysis of multiplex qPCR.**

The detection specificity analysis of multiplex triple (A) and double qPCR (B) is shown. In the specificity test of the multiplex qPCR method, in the MHV, MPV, and REO3 triple qPCR, no specific amplification curves were observed for MCMV, VSV, SeV, PVM, and NTC(Figure S3A); in the SeV and PVM double qPCR, no specific amplification curves were observed for MCMV, MHV, MPV, REO3, and NTC(Figure S3B). This indicates that the qPCR methods has good specificity.
